# Lymphopenia confers poorer prognosis in Myelodysplastic Syndromes with very low and low IPSS-M

**DOI:** 10.1038/s41408-023-00965-w

**Published:** 2023-12-21

**Authors:** David Fandrei, Tony Huynh, Marie Sébert, Lorea Aguinaga, Valeria Bisio, Rathana Kim, Emmanuelle Clappier, Marion Espéli, Karl Balabanian, Hélène Moins-Teisserenc, Antoine Toubert, Nicolas Dulphy, Pierre Fenaux, Lionel Adès, Lin-Pierre Zhao

**Affiliations:** 1 Université Paris Cité, Assistance Publique des Hôpitaux de Paris (APHP) Nord, Hôpital Saint-Louis, Hématologie Seniors, Paris, France; 2 Université Paris Cité, INSERM UMR 1160, Institut de Recherche Saint-Louis, Paris, France; 3 Université Paris Cité, INSERM UMR 944, Institut de Recherche Saint-Louis, Paris, France; 4 Université Paris Cité, Assistance Publique des Hôpitaux de Paris (APHP) Nord, Hôpital Saint-Louis, Laboratoire d’Hématologie, Paris, France; 5 Université Paris Cité, Assistance Publique des Hôpitaux de Paris (APHP) Nord, Hôpital Saint-Louis, Laboratoire d’Immunologie et Histocompatibilité, Paris, France

**Keywords:** Myelodysplastic syndrome, Cancer genetics

**To the editor:**

Myelodysplastic neoplasms (MDS) are hematopoietic stem cell (HSC) disorders arising in the bone marrow (BM) from clonal outgrowth, with the successive acquisition of genetic lesions. MDS are characterized by ineffective hematopoiesis leading to persistent cytopenia affecting one or more hematopoietic lineages, dysplasia, and an increased risk of transformation to acute myeloid leukemia (AML) [1]. Recent incorporation of genomic profiling in addition to hematologic and cytogenetic parameters in MDS prognostic assessment has improved patient risk stratification [2].

Along with MDS cell-intrinsic genetic lesions, increasing evidence indicates dynamic changes in both adaptive and innate immunity during MDS course, including T lymphocyte aberrant polarization [3], and reduced B [4] and NK [5] numbers and phenotype. The central role of host immunity in MDS pathogenesis is not captured by currently used revised or molecular international prognostic scoring systems (IPSS-R/M) [2, 6].

Absolute lymphocyte count (ALC) has been shown to predict inferior survival in solid cancers [7], and more recently, in the context of essential thrombocythemia based on a 4-tiered model incorporating age and absolute neutrophil count (ANC) [8]. In MDS, few reports have suggested a prognostic impact of ALC at diagnosis in del(5q) [9], non-del(5q) [10] and MDS with ring sideroblasts (RS) [11] patient subsets, mostly in IPSS-R-assessed low-risk (LR) patients [12]. However, these studies did not analyze the correlation between ALC and MDS genetic alterations, and it remains unclear if the addition of ALC in disease risk assessment may improve patient stratification in light of the most recent molecular prognostic scores [2]. In this work, we describe the prevalence of lymphopenia in MDS, the interaction with molecular features, and the prognostic impact of lymphopenia on outcome.

We included in this retrospective study all patients with a diagnosis of MDS according to WHO 2016 criteria, followed in our center (Saint-Louis Hospital, Paris, France) between January 2015 and January 2022, and who had at least 1 molecular evaluation using next-generation sequencing targeting a panel of 80 genes (Table S1) as previously described [13]. In our cohort, although the receiver operating characteristic plot-based optimal cutoff point was 1.6 G/L, we chose a close institutional laboratory reference of 1.5 G/L to define lymphopenia as more clinically relevant. Patients with ALC > 5 G/L (*n* = 2) at MDS diagnosis were excluded to rule out potential concomitant lymphoproliferative disorder. Low (LR), intermediate (IR), and high-risk (HR) MDS were defined according to IPSS-M (LR: IPSS-M Low and Very Low, scores <0.5; IR: IPSS-M Moderate Low and Moderate High, scores −0.5 to 0.5; and HR: IPSS-M High and Very High, scores > 0.5 respectively) and IPSS-R (LR: Very Low and Low; IR: Intermediate; HR: High and Very High).

Survival analyses were estimated between MDS diagnosis until death from any cause (OS) or last follow-up, or until AML transformation (leukemia-free survival, LFS) or last follow-up. Allogeneic stem cell transplantation (allo-SCT) was considered a censoring event. Cox proportional hazard regression model was applied for multivariate analysis. The discriminatory power of prognostic models and the relative fitness for the predictive score were evaluated using Harrel’s concordance index [14]. The study was performed in accordance with the ethical guidelines of the Declaration of Helsinki. All patients provided informed consent for molecular and clinical data analysis.

Our cohort included a total of 372 MDS patients (45% females) with a median age of 72 _range_[21–95] years, encompassing 230 (62%), 70 (19%), and 72 (19%) patients with IPSS-M LR, IR and HR MDS respectively (Table 1). Compared to IPSS-R, IPSS-M reclassified 175 patients (47%, including 97 (26%) who were upstaged and 78 (21%) downstaged, Fig. S1).

**Table 1:** Cohort characteristics according to the presśence or not of lymphopenia.

	Total (*N* = 372)	No Lymphopenia (ALC ≥ 1.5 G/L, *N* = 107)	Lymphopenia (ALC < 1.5 G/L, *N* = 265)	*p* value
Female gender (%)	168 (45%)	56 (52%)	112 (42%)	0.23
Age (years, median [range])	72 [21–95]	71 [21–95]	73 [28–95]	0.1
Hemoglobin (g/dL, median [IQR])	10.5 [9.1–12.0]	10.8 [9.3–12.0]	10.3 [9.1–12.0]	0.43
Platelets (G/L, median [IQR])	137 [76–230]	145 [80–300]	133 [73–210]	0.09
WBC (G/L, median [IQR])	4.10 [2.8, 5.8]	5.38 [4.0, 7.7]	3.50 [2.4, 5.0]	**<0.01**
Monocytes (G/L, median [IQR])	0.34 [0.18–0.53]	0.42 [0.25–0.78]	0.31 [0.17–0.46]	**0.04**
Lymphocytes (G/L, median [IQR])	1.20 [0.87–1.60]	1.90 [1.6–2.20]	1.01 [0.77–1.30]	**<0.01**
Neutrophils (G/L, median [IQR])	2.10 [1.20–3.70]	2.70 [1.60–4.20]	1.90 [1.10–3.40]	**0.01**
BM Blasts (%, median [IQR])	3.00 [2.0–4.0]	3.00 [2.0–4.0]	3.00 [2.0–5.0]	0.56
WHO 2016 Category				
MDS without EB/without RS	186 (50%)	54 (51%)	132 (49%)	0.82
MDS-SLD	43 (12%)	12 (11%)	31 (12%)	
MDS-MLD	143 (38%)	42 (39%)	101 (38%)	
MDS-RS without EB	49 (13%)	22 (21%)	27 (10%)	**<0.01**
MDS-RS-MLD	29 (8%)	17 (16%)	12 (5%)	
MDS-RS-SLD	20 (5%)	5 (5%)	15 (6%)	
Isolated Del5q	26 (7%)	5 (5%)	21 (8%)	0.37
MDS-EB	99 (27%)	21 (20%)	78 (29%)	0.09
MDS-EB1	62 (17%)	11 (10%)	51 (19%)	
MDS-EB2	37 (10%)	10 (9%)	27 (10%)	
MDS-U	12 (3%)	5 (5%)	7 (3%)	0.33
IPSS-R				
Low	243 (65%)	77 (72%)	166 (63%)	0.05
Intermediate	69 (19%)	15 (14%)	54 (20%)	0.24
High	60 (16%)	15 (14%)	45 (17%)	0.64
IPSS-M				
Low	230 (62%)	74 (69%)	156 (59%)	**0.03**
Intermediate	70 (19%)	15 (14%)	55 (21%)	0.19
High	72 (19%)	18 (17%)	54 (20%)	0.54

*BM* bone marrow, *EB* excess of blasts, *IPSS* international prognostic scoring system, *MDS* myelodysplastic syndromes, *RS* ring sideroblasts, *SLD* single lineage dysplasia, *WBC* while blood cell count, *WHO* world health organization.

Bold values denote statistical significance at the *p* < 0.05 level.

**Fig. 1:**
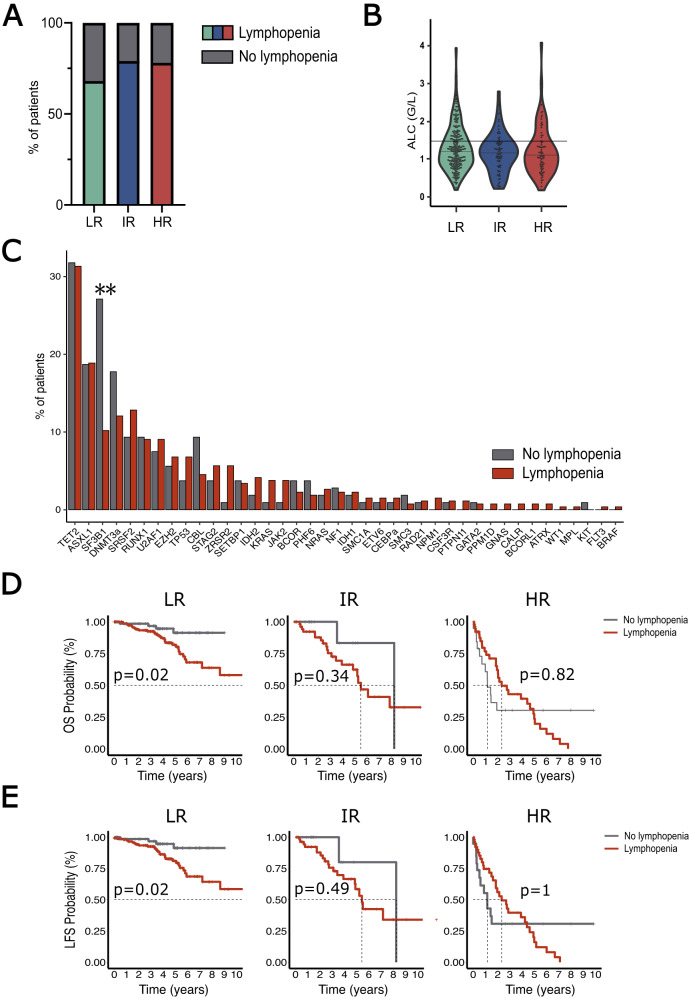
Interaction of lymphopenia with MDS clinical and molecular features, and prognostic impact. **A** Prevalence of lymphopenia (defined as absolute lymphocyte count (ALC) < 1.5 G/L) in IPSS-M subgroups; (**B**) ALC in IPSS-M subgroups (threshold of 1.5 G/L is indicated with a black bar); (**C**) Mutational landscape of most commonly mutated genes in MDS patients with or without lymphopenia; **p < 0.01; (**D**) Impact of lymphopenia on MDS patients’ overall survival (OS) according to IPSS-M strata; (**E**): Impact of lymphopenia on MDS patients’ leukemia-free survival (LFS) according to IPSS-M strata.

The median ALC in our whole cohort was 1.20 _IQR_[0.87–1.60] G/L, and there was a trend toward lower ALC in patients aged above 65 years (1.28 G/L _IQR_[0.92–1.68] versus 1.15 G/L _IQR_[0.84–1.53] for patients below or above 65 years respectively, *p* = 0.08, Fig. S2A) and in male patients (1.28 G/L _IQR_[0.95–1.66] versus 1.1 G/L _IQR_[0.84–1.5] in female and male patients respectively, *p* = 0.09, Fig. S2B), although not statistically significant. Lymphopenia was highly prevalent in all MDS subgroups (68%, 79 and 75% in LR, IR and HR MDS respectively) (Fig. 1A, B), and ALC inversely correlated with both IPSS-M (Spearman rho = −0.13, *p* = 0.01; Fig. S2C) and IPSS-R (Spearman rho = −0.10, *p* = 0.04; Fig. S2D) scores.

We next compared MDS patients with (*n* = 265, 71%) and without (*n* = 107, 29%) lymphopenia (Table 1). The presence of lymphopenia correlated with more severe other cytopenias, including lower white blood cell (WBC) count (median 3.50 [2.4, 5.0] versus 5.38 [4.0, 7.7], *p* < 0.01), lower absolute neutrophil (median 1.90 _IQR_[1.10–3.40] versus 2.70 _IQR_[1.60–4.20], *p* = 0.01) and monocyte (median 0.31 _IQR_[0.17–0.46] versus 0.42 _IQR_[0.25–0.78], *p* = 0.04) counts. There was no difference in the representation of MDS with excess of blast (EB) subtypes (78/265, 29% versus 21/107, 21% in patients with or without lymphopenia respectively; *p* = 0.9). However, we found significantly fewer MDS patients with ring sideroblasts (RS) subsets in the lymphopenia group (27/265, 10% versus 22/107, 21%; *p* < 0.01).

In our whole cohort, 332/372 (89%) of patients had at least one molecular abnormality (Fig. 1C). Lymphopenia was not significantly associated with any recurrently mutated pathways, including epigenetic regulation (*TET2, IDH1/2, DNMT3A, ASXL1, EZH2, BCOR*; OR 1.01 [0.64–1.59]), spliceosome (*SF3B1, SRSF2, U2AF1, ZRSR2*; OR: 0.77 [0.49–1.22]), signal transduction (*NRAS, KRAS, KIT, NK1, CBL, NF1*; OR: 1.22 [0.65–2.29]), transcription regulation (*WT1, RUNX1, GATA2, ETV6*; OR: 1.33 [0.65–2.73]), cohesin complex (*STAG2*, *RAD21*, *SMC3*: OR 0.96 [0.43–2.15]), and DNA repair (*TP53, PPM1D*; OR: 1.33 [0.65–2.73]) (Fig. S3). Of note, the prevalence of *SF3B1* mutation (29/107 (27%) versus 27/265 (10%), *p* < 0.01) was higher in MDS patients without lymphopenia (Fig. 1C).

Next, we estimated the probability of OS and LFS across IPSS-M categories and analyzed the impact of lymphopenia on patient outcome. Median follow-up of the entire cohort was 49.6 _IQR_[20.3–55.0] months. IPSS-M categories showed significantly different probabilities on both OS (Fig. S4A) and LFS (Fig. S4B) (both *p* < 0.01), and this effect was maintained in a multivariate model including age and sex as covariate (HR 2.24 _95%CI_[1.91–2.62], *p* < 0.01 for OS; HR 2.27 _95%CI_[1.94–2.67], *p* < 0.01 for LFS). Across different IPSS-M categories, lymphopenia at MDS diagnosis adversely impacted OS in LR MDS patients (median 8.76 _95%CI_[NE-NE] years versus not reached, *p* = 0.02) but not in IR (median 5.50 _95%CI_[4.86-NE] versus 8.24 _95%CI_[NE-NE] years, *p* = 0.34) or in HR (median 2.33 _95%CI_[1.88–4.93] versus 1.42 _95%CI_[0.70-NE] years in MDS with or without lymphopenia respectively, *p* = 1) patients (Fig. 1D). Similarly, the presence of lymphopenia at MDS diagnosis significantly impacted LFS only in LR-MDS (median 12.30 _95%CI_[8.67-NE] years versus not reached, *p* = 0.02; Fig. 1D). In the LR-MDS subgroup, the further inclusion of sex, age at MDS diagnosis and thrombocytopenia (variables significantly impacting OS and LFS in univariate analysis) in a multivariate model resulted in borderline *p*-values regarding the impact of lymphopenia on OS (*p* = 0.06; Table S2) and LFS (*p* = 0.05; Table S3). We then estimated the impact of lymphopenia in *SF3B1*-mutated MDS patients, given its association with ALC. In this subgroup, the presence of lymphopenia also adversely impacted patient outcome regarding OS (Fig. S5A) or LFS (Fig. S5B) according to IPSS-M strata.

Finally, we wondered if lymphopenia as an additional parameter might enhance current prognostic stratification models. While IPSS-M score alone refined Harrel’s concordance index compared to IPSS-R (0.76 _95%CI_[0.70–0.82] versus 0.72 _95%CI_[0.66–0.78] for both OS and LFS), the addition of lymphopenia variable did not significantly improve each risk model’s stratification performance (OS: 0.77 _95%CI_[0.71–0.83] versus 0.76 _95%CI_[0.70–0.82]; LFS: 0.73 _95%CI_[0.67–0.80] versus 0.72 _95%CI_[0.66–0.78] in IPSS-R/M with or without lymphopenia respectively; Fig. S6).

In this single-center retrospective analysis, using a well-clinically characterized cohort with molecular annotations, in which we validated IPSS-M prognostic value, lymphopenia was highly prevalent at MDS diagnosis in all IPSS-M subgroups and ALC negatively correlated with disease severity.

To define lymphopenia, we used a local laboratory-based threshold of 1.5 G/L higher than previously used cut-offs [10, 12, 15]. This yielded a high prevalence (more than 2 thirds) of lymphopenia across all IPSS-M subgroups, possibly suggesting a specific effect of MDS on ALC. Indeed, although ALC is known to decrease with age (due to thymic involution or myeloid skewing), mean ALC remains >2 G/L throughout life in healthy individuals [16]. The absence of difference in ALC according to age or gender in our cohort could corroborate this hypothesis. The relative bone marrow failure and increased myeloid bias secondary to specific MDS-related somatic mutations in HSC [17] likely contribute to this phenotype. Consistently, lymphoid progenitors [4] and mature lymphocytes [18] have been reported as decreased in MDS patients. Of note, *SF3B1*-mutated MDS patients had a decreased prevalence of lymphopenia, confirming previous reports showing the relative preservation of ALC in MDS-RS subtypes [10, 12], in line with preferential erythroid-restricted alterations in this MDS subgroup.

The presence of lymphopenia adversely impacted OS in the LR-MDS subgroup but not in IR and HR patients, suggesting a protective role of host immunity on disease progression mostly prevalent at disease onset. In more advanced MDS stages, other alterations as cell-intrinsic genetic alterations or BM microenvironment modifications may have a stronger impact in driving disease progression, potentially explaining the lack of significant stratification benefit in adding lymphopenia variable in both IPSS-R and IPSS-M scores. This hypothesis is corroborated by the restricted prognostic impact of lymphopenia on LFS in LR-MDS subgroup. Of note, the association between lymphopenia and survival in both *SF3B1*-mutated and non-mutated MDS patients confirm previous results from other groups [11, 12]. Additional studies are needed to decipher whether or not lymphopenia may result from clone-intrinsic defective lymphopoiesis, or may contribute ro the worsening of cytopenia, the latter hypothesis suggesting a potential benefit of immunomodulatory drugs.

Given the retrospective setting of our study, we cannot exclude certain confounding factors that might have affected ALC at diagnosis, as concomitant infections or treatment with steroids.

Future more in-depth works will be needed to better correlate lymphocyte subsets, multiparametric phenotyping, and MDS genotype, potentially leading to targeted immunotherapies, especially in the LR-MDS subgroup.

### Supplementary information

Supplemental Material
