# Supplementary material for: Lymphopenia confers poorer prognosis in Myelodysplastic Syndromes with very low and low IPSS-M
Source: Blood Cancer J. 2023 Dec 21;13(1):193. doi: 10.1038/s41408-023-00965-w (PMC10733334; doi:10.1038/s41408-023-00965-w)
Supplement: Supplementary file 1 — Supplemental Material [file 41408_2023_965_MOESM1_ESM.docx]

**Supplemental material**

**Title:** Lymphopenia confers poorer prognosis in Myelodysplastic Syndromes with very low and low IPSS-M

**Supplemental Figure 1:** Prognostic restratification from IPSS-R to IPSS-M.

**Supplemental Figure 2:** Lymphopenia association with patients’ characteristics.

**Supplemental Figure 3:** Forest plot showing the association of commonly mutated pathways with lymphopenia.

**Supplemental Figure 4:** Overall survival (OS) and leukemia-free survival (LFS) according to grouped IPSS-M categories.

**Supplemental Figure 5:** Overall (**A**) and leukemia-free (**B**) survival in *SF3B1*-mutant MDS patients with or without lymphopenia according to IPSS-M strata.

**Supplemental Figure 6:** Harrel’s concordance index regarding overall survival (OS) and leukemia-free survival (LFS) for IPSS-R and IPSS-M scores with or without lymphopenia.

**Supplemental Table 1:** List of 80 genes for targeted next-generation sequencing.

**Supplemental Table 2:** Univariate and multivariate Cox regression model for overall survival in IPSS-M low-risk MDS patients (n=230).

**Supplemental Table 3:** Univariate and multivariate Cox regression model for leukemia-free survival in IPSS-M low-risk MDS patients (n=230).

**Supplemental Figure 1:** Prognostic restratification from IPSS-R to IPSS-M. A total of 175 (47%) patients were reclassified, including 97 (26%) who were upstaged and 78 (21%) downstaged.

**
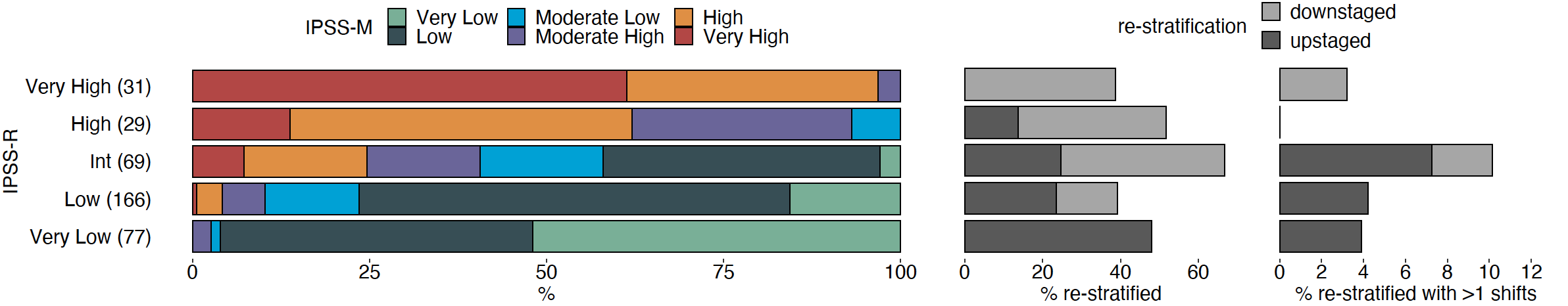
**

**Supplemental Figure 2:** Lymphopenia association with patients’ characteristics. **A**: absolute lymphocyte count (ALC) according to age; **B**: ALC according to gender; **C**: univariate linear regression between ALC and IPSS-M score; **D**: univariate linear regression between ALC and IPSS-R score


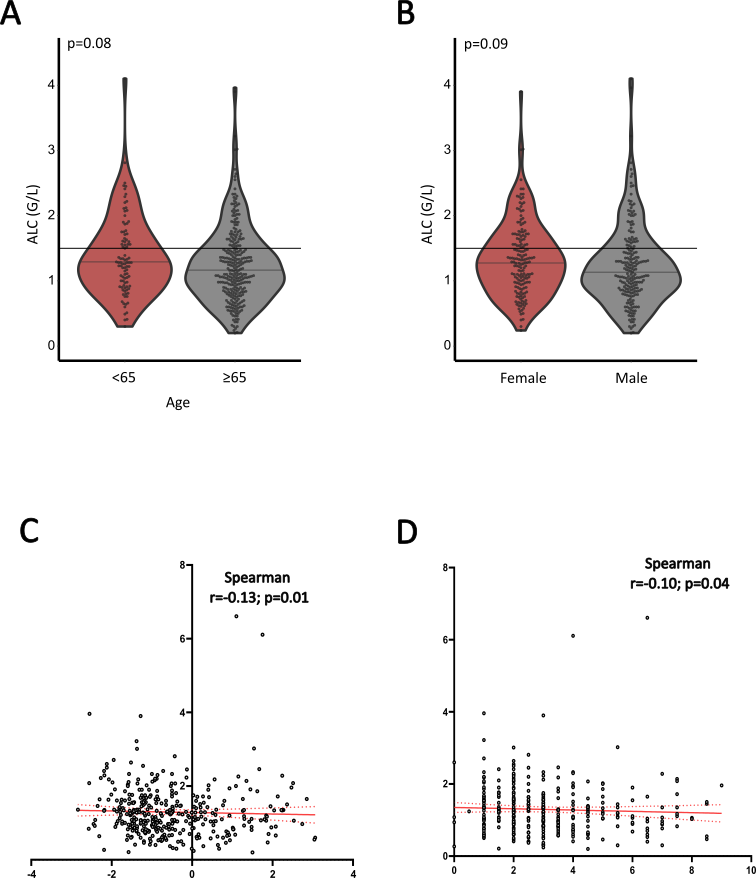


**Supplemental Figure 3:** Forest plot showing the association of commonly mutated pathways with lymphopenia.

*Epigenetic regulators: TET2, IDH1/2, DNMT3A, ASXL1, EZH2, BCOR; cohesion complex: STAG2, RAD21, SMC3; splicing factors: SRSF2, U2AF1, ZRSR2; excluding SF3B1; DNA repair: TP53, PPM1D; signaling: NRAS, KRAS, KIT, NK1, CBL, NF1; transcription factors: WT1, RUNX1, GATA2, ETV6. Odds ratio* ≤1 and >1 are indicated in blue and red respectively.


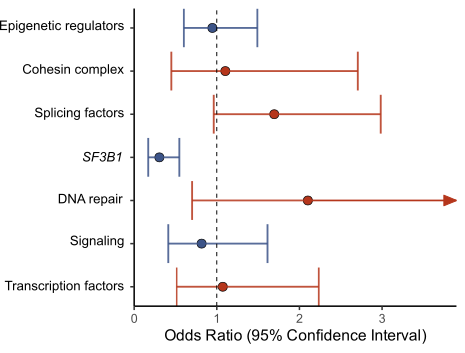


**Supplemental Figure 4:** (**A**) Overall survival (OS) and (**B**) leukemia-free survival (LFS) according to grouped IPSS-M categories.

*LR: IPSS-M Low and Very Low, scores <0.5; IR: IPSS-M Moderate Low and Moderate High, scores -0.5 to 0.5; HR: IPSS-M High and Very High, scores >0.5*

*
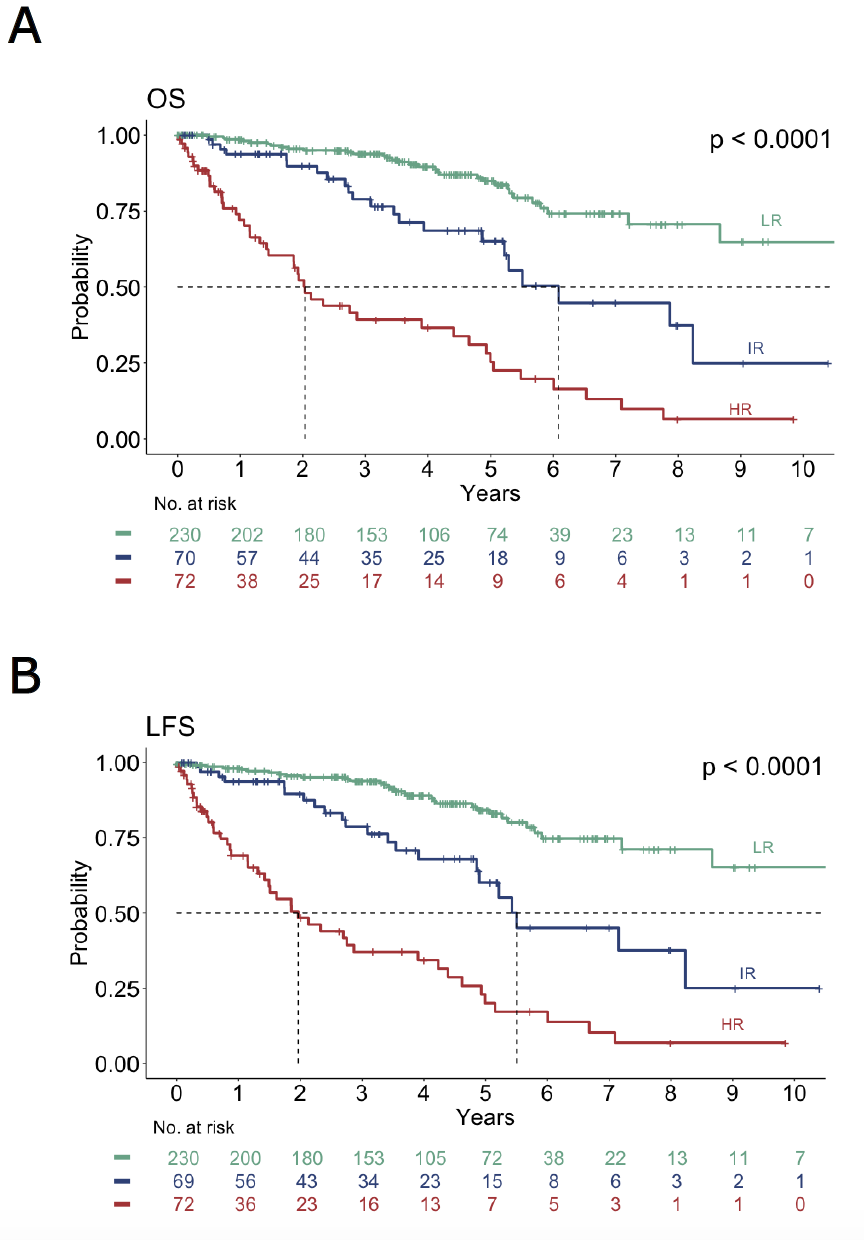
*

**Supplemental Figure 5:** (**A**) Overall survival (OS) and (**B**) leukemia-free survival (LFS) in *SF3B1*-mutant MDS patients with or without lymphopenia according to IPSS-M strata.

**
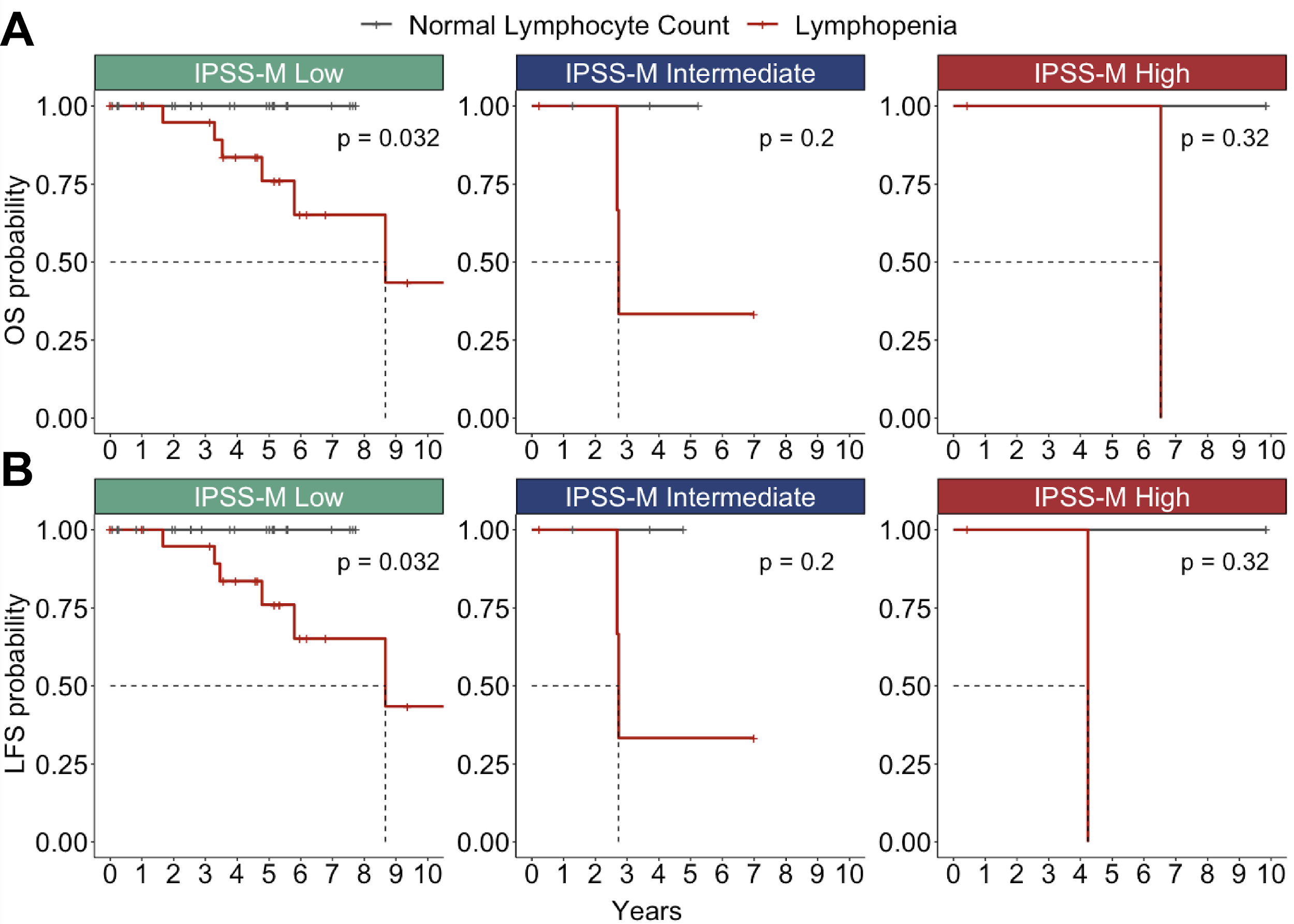
**

**Supplemental Figure 6:** Harrel’s concordance index regarding overall survival (OS) and leukemia-free survival (LFS) for IPSS-R and IPSS-M scores with or without lymphopenia.

**
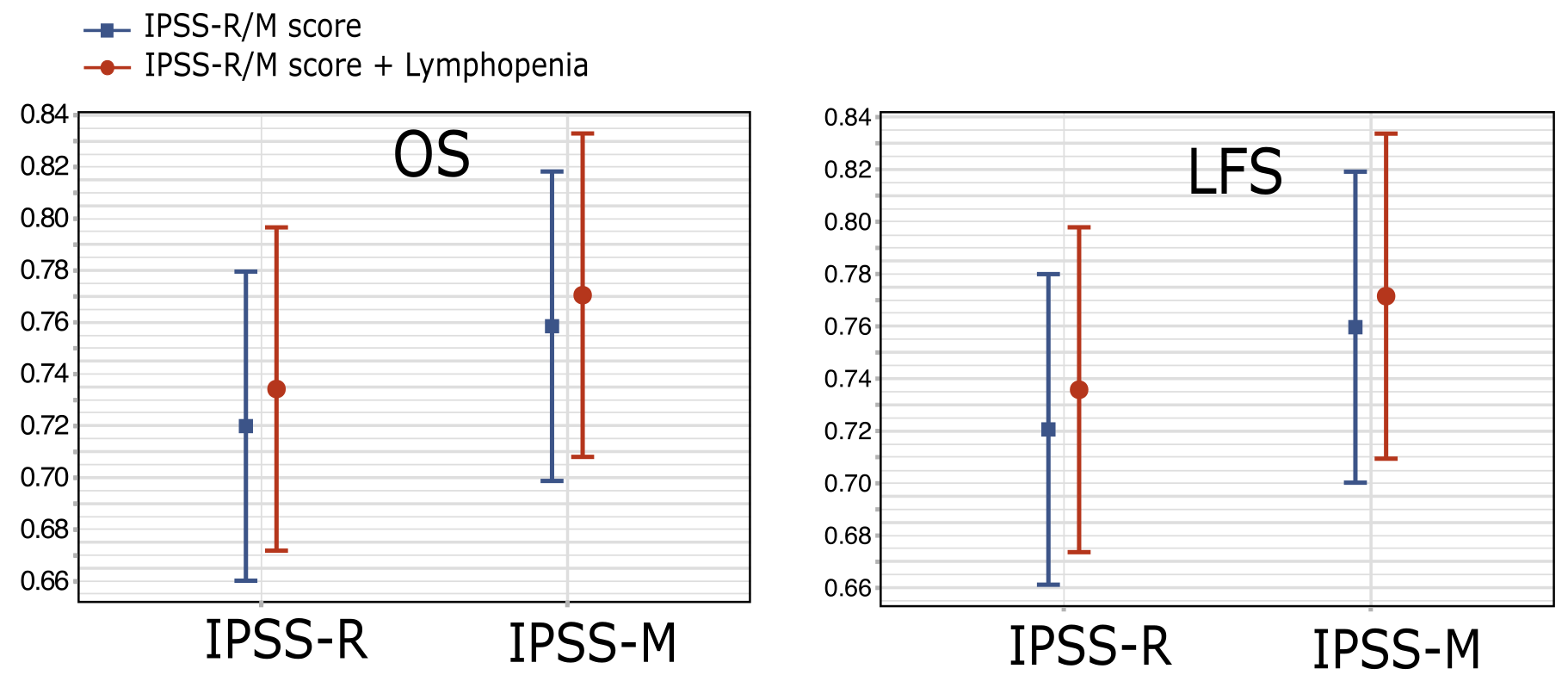
**

**Supplemental Table 1:** List of 80 genes for targeted next-generation sequencing. *A panel of 80 genes commonly analyzed in myeloid malignancies was examined using next-generation sequencing (NGS) on an Illumina platform (Illumina, San Diego, CA, USA). Libraries were prepared from 200 ng of DNA using a custom probes panel for the capture of all coding sequences (SureSelectXT Target Enrichment System, Agilent, Santa Clara, CA, USA). Variant calling was performed using an in-house pipeline. High-probability pathogenic mutations were retained based on the available databases for SNPs, somatic mutations in cancer, prediction algorithms, and frequencies of variant read.*

| **gene** | **reference** | **gene** | **reference** | **gene** | **reference** | **gene** | **reference** |
| --- | --- | --- | --- | --- | --- | --- | --- |
| ASXL1 | NM_015338 | DDX41 | NM_016222 | KMT2A/MLL | NM_001197104 | RPL5 | NM_000969 |
| ASXL2 | NM_018263 | DKC1 | NM_0001363.2 | KMT2D/MLL2 | NM_003482 | RTEL1 | NM_032957 |
| ATM | NM_000051 | DNAJC21 | NM_001012339 | KRAS | NM_033360 | RUNX1 | NM_001754 |
| ATRX | NM_000489 | DNMT3A | NM_022552 | LIG4 | NM_002312.3 | SAMD9 | NM_017654 |
| BCOR | NM_017745 | EP300 | NM_001429 | MDM4 | NM_002393 | SAMD9L | NM_152703 |
| BCORL1 | NM_021946 | ERCC6L2 | NM_001010895 | MECOM | NM_001105078 | SBDS | NM_016038 |
| BRAF | NM_004333 | ETNK1 | NM_018638 | MPL | NM_005373 | SETBP1 | NM_015559 |
| BRCA1 | NM_007294 | ETV6 | NM_001987 | MYC | NM_002467 | SF1 | NM_004630 |
| BRCA2 | NM_000059 | EZH2 | NM_004456 | NF1 | NM_001042492 | SF3B1 | NM_012433 |
| BRCC3 | NM_024332 | FLT3 | NM_004119 | NPM1 | NM_002520 | SMC1A | NM_006306 |
| CALR | NM_004343 | GATA2 | NM_032638 | NRAS | NM_002524 | SMC3 | NM_005445 |
| CBL | NM_005188 | GSKIP | NM_001271904 | PARN | NM_002582 | SRP72 | NM_006947 |
| CDAN1 | NM_138477 | HRAS | NM_005343 | PHF6 | NM_001015877 | SRSF2 | NM_003016 |
| CEBPA | NM_004364 | IDH1 | NM_005896 | PPM1D | NM_003620 | STAG2 | NM_001042749 |
| CHEK2 | NM_007194.3 | IDH2 | NM_002168 | PRPF8 | NM_006445 | TET2 | NM_001127208 |
| CREBBP | NM_004380 | IRF1 | NM_002198 | PTEN | NM_00314 | TP53 | NM_001126112 |
| CSF3R | NM_156039 | JAK2 | NM_004972 | PTPN11 | NM_002834 | U2AF1 | NM_006758 |
| CTC1 | NM_02599 | KDM5A | NM_001042603 | RAD21 | NM_006265 | U2AF2 | NM_007279 |
| CTCF | NM_006565 | KDM6A | NM_021140 | RIT1 | NM_006912 | WT1 | NM_024426 |
| CUX1 | NM_001913 | KIT | NM_000222 | RPL11 | NM_000975 | ZRSR2 | NM_005089 |

**Supplemental Table 2:** Univariate and multivariate Cox regression model for overall survival in IPSS-M low-risk MDS patients (n=230).

*BM: bone marrow.*

|  |  |  |  |  |  |  |
| --- | --- | --- | --- | --- | --- | --- |
|  | **OS in LR MDS (n=230)** | | | | | |
|  | **Univariate** | | | **Multivariate** | | |
|  | **HR** | **95% CI** | **p-value** | **HR** | **95% CI** | **p-value** |
| Sex (male) | 2.83 | 1.31-6.14 | **<0.01** | 3.04 | 1.38-6.7 | **<0.01** |
| Age at diagnosis (years) | 1.05 | 1.01-1.09 | **<0.01** | 1.05 | 1.01-1.09 | **<0.01** |
| Anemia <10 g/dl | 1.37 | 0.68-2.76 | 0.38 |  |  |  |
| Neutrophiles >1.8G/l | 0.86 | 0.42-1.74 | 0.67 |  |  |  |
| Thrombopenia <100G/l | 0.21 | 0.05-0.87 | **0.03** | 0.17 | 0.04-0.72 | **0.02** |
| BM-Blasts >5% | 2.71 | 0.64-11.4 | 0.17 |  |  |  |
| Lymphopenia <1.5G/l | 3.13 | 1.10-8.96 | **0.02** | 2.81 | 0.98-8.1 | 0.06 |
| Lymphocyte count | 0.82 | 0.43-1.59 | 0.56 |  |  |  |

**Supplemental Table 3:** Univariate and multivariate Cox regression model for leukemia-free survival in IPSS-M low-risk MDS patients (n=230).

*BM: bone marrow.*

|  | **LFS in LR MDS (n=230)** | | | | | |
| --- | --- | --- | --- | --- | --- | --- |
|  | **Univariate** | | | **Multivariate** | | |
|  | **HR** | **95% CI** | **p-value** | **HR** | **95% CI** | **p-value** |
| Sex (male) | 2.85 | 1.32-6.19 | **<0.01** | 3.04 | 1.38-6.71 | **<0.01** |
| Age at diagnosis (years) | 1.05 | 1.01-1.09 | **<0.01** | 1.05 | 1.01-1.09 | **<0.01** |
| Anemia <10 g/dl | 1.37 | 0.679-2.78 | 0.3773 |  |  |  |
| Neutrophiles >1.8G/l | 0.89 | 0.44-1.81 | 0.7463 |  |  |  |
| Thrombopenia <100G/l | 0.21 | 0.04-0.86 | **0.03** | 0.17 | 0.04-0.71 | **0.02** |
| BM-Blasts >5% | 2.64 | 0.62-11.1 | 0.1862 |  |  |  |
| Lymphopenia <1.5G/l | 3.2 | 1.12-9.19 | **0.02** | 2.92 | 1.01-8.39 | **0.05** |
| Lymphocyte count | 0.81 | 0.42-1.55 | 0.5217 |  |  |  |
